# End-of-life care in multiple system atrophy: UK survey of patients and families

**DOI:** 10.1136/spcare-2024-005045

**Published:** 2024-08-13

**Authors:** David Oliver, Andy Barrick, Christopher Kobylecki, Jalesh Panicker, Niall Quinn, Emma Rushton, Anette Schrag, Karen Walker, Kailash Bhatia

**Affiliations:** 1 Tizard Centre, University of Kent, Canterbury, UK; 2 Multiple System Atrophy Trust, London, UK; 3 Department of Neurology, Manchester Centre for Clinical Neurosciences, Northern Care Alliance NHS Foundation Trust, Salford, Greater Manchester, UK; 4 Division of Neuroscience, Manchester Academic Heath Science Centre, University of Manchester, Manchester, UK; 5 Department of Uro-neurology, National Hospital for Neurology and Neurosurgery, London, UK; 6 Faculty of Brain Sciences, University College London Queen Square Institute of Neurology, London, UK; 7 University College London Queen Square Institute of Neurology, London, UK; 8 Institute of Neurology, National Hospital for Neurology and Neurosurgery, London, UK; 9 Department of Clinical and Movement Neurosciences, University College London, London, UK

**Keywords:** Advance Directives, Chronic conditions, Palliative Care, Terminal care, Quality of life

## Abstract

**Objectives:**

People with multiple system atrophy (MSA) and their carers may have many concerns about their disease and the future. This survey of people with MSA and their carers aimed to increase understanding of end-of-life care and palliative care for this group.

**Methods:**

A survey was undertaken by the MSA Trust of people living with MSA and carers of those with the condition between August and October 2022.

**Results:**

520 people responded: 215 people with MSA, 214 carers and 91 former carers. The modal class for age in people with MSA was 65–74 years, with 52% male. 76% of people living with MSA had thought to some extent about what they wanted to happen towards the end of their lives. 38% of respondents had discussed end-of-life care options with a healthcare professional and of those who had, over 81% found the conversation helpful. Nevertheless, for 37% of former carers, the death had been unexpected. Only a minority of people living with MSA had been referred for specialist palliative care. 65% of the former carers reported that they were satisfied with the quality of end-of-life care.

**Conclusion:**

People with MSA and their carers continue to face many complex physical and emotional issues that would benefit from palliative care. Discussions about care at the end of life were generally perceived as helpful, but although the deterioration was often discussed, many families seemed unprepared for the death. Palliative care services were involved but this appeared limited.

**WHAT IS ALREADY KNOWN ON THIS TOPIC**

- People with multiple system atrophy (MSA) have many issues that could benefit from palliative care.
- Palliative care may be limited for people with MSA.

**WHAT THIS STUDY ADDS**

- People with MSA would often wish to discuss end-of-life issues.
- Palliative care was acceptable to people with MSA but was often limited.

**HOW THIS STUDY MAY AFFECT RESEARCH, PRACTICE OR POLICY**

- Education and awareness of the professional teams are needed.
- Palliative care is appropriate early in the disease progression.

## Introduction

Multiple system atrophy (MSA) is a sporadic, rapidly progressive neurodegenerative disease that presents with aspects of parkinsonism, cerebellar ataxia and autonomic dysfunction.^1^ The incidence is 1.9–4.9/100 000 persons and the age-adjusted prevalence is 4.4/100 000 persons.^1^ Neuropathological changes, including alpha-synuclein-positive oligodendrocytic cytoplasmic inclusions, are found in brain areas involved in movement and autonomic control.^2^

Two main clinical subtypes are described: the MSA-parkinsonian variant, characterised pathologically by striatonigral degeneration and clinically by limb bradykinesia and tremor and the MSA-cerebellar variant, characterised pathologically by olivopontocerebellar atrophy and clinically by ataxia, nystagmus and scanning dysarthria.^1 2^ However, there may be a mixed picture, clinically and pathologically, and autonomic dysfunction is common to both subtypes, along with postural hypotension, supine hypertension, urinary and bowel symptoms, and sexual dysfunction.^2^ Speech and swallowing disturbances are also common in both subtypes. There are many symptoms requiring complex management and there is variable progression, with a median survival from symptom onset of 7–9 years.^1 3^

Palliative care has been suggested for people with neurological disease for many years,^4^ and there is increasing evidence that early involvement may help in supporting quality of life, reducing symptoms and supporting both the patient and family.^5^ However, palliative care is less often discussed in MSA, and it has been suggested that this may be due to a reluctance on the part of healthcare professionals (HCPs) and social care professionals to discuss the issues of deterioration and dying and the association with the end of life, causing distress to patients and families.^6^ The previous MSA Trust 2019 Needs Survey did show that there were many needs for people with MSA and their families, and a substantial proportion of participants had received support from palliative care services.^3 6^ A survey of European neurologists and palliative care specialists showed that collaboration in the care of people with MSA was uncommon, with only 20% of palliative care specialists and 32% of neurologists reporting strong or moderate collaboration, compared with 60%–70% for amyotrophic lateral sclerosis/motor neuron disease (MND) and cerebral tumours.^7^

Studies have shown that patients vary in their preferences in the discussion of end of life, and although 63% had discussed these issues with a family member or a lawyer, primarily in completing a power of attorney or advance directives, the discussion with HCPs was much lower.^8^ However, when asked in a survey, over 50% expressed a desire for discussion on end-of-life care.^8^ There was also evidence that those with longer disease duration and whose quality of life and independence were more impacted were more likely to discuss advance care planning (ACP).^9^

The MSA Trust, a charity that supports people with MSA in the UK and Ireland, has undertaken a survey of people with MSA and their carers. This survey included questions on end-of-life and palliative care, and the results are presented here.

## Methods

Three anonymous questionnaires were developed: for people with MSA, current carers of someone with MSA and former carers of someone who had died with MSA. The questionnaires were modelled on a previous Needs Survey in 2019^3^ and had been developed in conjunction with MSA Trust specialists, nurses, social welfare specialists, neurologists, palliative care professionals and people living with MSA, and current and former carers. The survey was developed by a company, IQVIA, and cognitive testing was undertaken with four people with MSA, two carers and two former carers. Several changes were made to develop the final version.

The questionnaire was sent as an online survey, to be completed anonymously, to the membership of the Trust: people with MSA, carers and former carers who had remained members of the Trust following the death within the last 3 years. There were checks undertaken to ensure that it was not sent to people who had died. The online survey was promoted through social media, and details were given in the MSA Trust magazine. A paper copy of the questionnaire was sent to members of the Trust, depending on consent preferences. It was also possible for people to complete the questionnaire over the telephone and in different languages if required. The online survey and the paper questionnaires were distributed between August 2022 and October 2022.

As this was part of a standard anonymised Needs Survey sent out by the MSA Trust every 3 years to its membership and was anonymous, ethical approval was not necessary. All responses were anonymised and kept confidential and secure.

## Results

A total of 1955 people were contacted about the survey: 933 people with MSA, 736 carers and 226 former carers. There were 520 responses, with an overall response rate of 27%—215 people with MSA (22%), 214 carers (29%) and 91 former carers (40%). Of the responses, 412 were online, 101 were on paper and 7 were obtained by other methods. Of the people with MSA, 161 (76%) had completed the questionnaire themselves, 32 (15%) were helped by family or friends and 20 (9%) were completed by HCPs. It was emphasised in the questionnaire that if other people completed the questionnaire, they should reflect the views of the person with MSA rather than their own views.

The respondents were primarily over 50 years old, and the modal class for age in people with MSA was 65–74 years, covering 40% of respondents, followed by 55–64 years with 29%, 6% aged 45–54, 69% were 55 to 74 and none less than 45 years. 52% of the people with MSA were male. 98% described their ethnicity as ‘white’. 104 (48%) of the people with MSA were female and 56 (62%) of the carers were female, primarily a spouse or partner and rarely a son/daughter (two, 1%). 96% of the carers were the main and only carers, and 98% lived with the person with MSA. Of the former carers, 88% had lived with the person until death, with 8 (9%) of the people with MSA dying in a hospice or nursing home.

Many people with MSA had experienced delays in diagnosis, with 49% taking over 2 years from their first symptoms. The most common diagnosis was Parkinson’s disease, which was the first diagnosis for 41%. 41% had not been given information about MSA at diagnosis, whereas 31% had received information produced by the MSA Trust. 44% would have liked to discuss planning for the future. There were many physical, psychological, emotional, social and financial issues that were faced.

### Discussion about end-of-life care

Many of the people with MSA had thought about what would happen at the end of their lives; 76% had considered these issues, whereas 22% had not. 66% of carers and 67% of former carers had discussed the future to some extent with the person with MSA as the disease advanced, whereas 32% had not. Many carers added their own comments, including:

We had previously discussed end of life care and he was clear that he did not want to prolong his life unnecessarily so when diagnosed it was easier to broach the subject.

Conversation with our neurologist who suggested an end-of-life care plan.

38% of people with MSA and 40% of carers reported that a discussion had taken place with an HCP. 63% of former carers had these discussions, and this higher level may be because the people living with MSA may be at an earlier stage in their disease progression, whereas former carers had coped until death. Of those who had had discussions, over 80% found this helpful; 81% were people with MSA, 85% were carers and 84% were former carers. One-third of respondents who had not had a discussion about palliative care or ACP would have liked to have these discussions, although 31% did not want to discuss these issues with a doctor.

### Involvement of specialist palliative care

Across all three groups, 34% had no involvement with specialist palliative care (hospice care services), which was involved with only a minority of people with MSA in this survey. Only 13% of people with MSA were using services, 41% felt that it was not required, and 11% had been involved but were no longer able to attend as the service had been stopped, had been limited in time or were no longer able to access it. 21% of carers were involved with services. Only a small minority—2% of people with MSA and 12% of carers—did not want any involvement. The services involved were primarily community-based: day hospice (51%), community palliative care team (38%) and outpatient clinic (16%), with only 16% having a hospice inpatient unit admission and 14% having a hospital palliative care team. Of those who had experienced specialist palliative care, 82% reported that the staff showed good knowledge of MSA.

### Satisfaction with end-of-life care

The majority of former carers (65%) expressed satisfaction with the care given at the end of life, but 15% were unhappy about the care. 21% felt that end-of-life care had not been required. Less than a third reported that the person did not die where they wished to be. This had often been due to issues with communication and speech or that following admission to the hospital, they were too ill to be moved.

There were comments about the possible areas of improvement. These included timely discussion and preparation for deterioration and death, and guidance and access to resources and help at the correct time, but without increasing stress on carers and family:

I feel that more timely discussions need to be held between health professionals and families about palliative care and end-of-life care. I planned these and discussed my wife’s wishes. I feel that health officials stay clear of such discussions for their own emotional well-being.

My wife spent 9 days in hospital before she died. This came as a shock, as nobody had prepared me for such an outcome. I don't believe I was given enough information by the staff until the morning she was dying, she was just another case on the ward.

### Advance care planning

Many people with MSA had made plans for the future, with 73% having a will, 58% having Lasting Power of Attorney (LPA) for Property and Finance, and 54% having an LPA for Health and Welfare. Other advance care plans were less common: the advance decision to refuse treatment (18%), do not attempt cardio-pulmonary resuscitation (30%), emergency care plan (15%), personalised end-of-life care plan (12%) and brain donation (12%). Over 60% of respondents felt that care plans were ‘not necessary’.

Carers and former carers had rarely completed an emergency care plan, which could have helped ensure care continued if they became unwell or incapacitated. 76% of carers and 70% of former carers had not completed one. Some carers had made arrangements with paid carers, care homes or families:

The plan has been agreed with…via a Carers' emergency support back up system plus card to be kept with me at all time. The plan has details of all medication, routines, key contact numbers, and links to my husband’s respect form completed with the guidance of our local hospice.

I was able to contact a local hospital neurology unit, which provided free planned respite on a number of occasions and also when I was admitted to hospital in emergency. My adult children were able to cover for a weekend until the respite was organised.

### Expectedness of death

Former carers were often not expecting the death; only 22% expected the death when it did occur; 41% were expecting the death but felt it occurred sooner than they had thought, and for 37%, the death was unexpected.

## Discussion

This survey is one of the largest internationally and provides an insight into the issues faced by people with MSA and their carers relating to end-of-life care. Although the response rate was only 27% overall, this compares favourably with other surveys of patients and families.^10^ The characteristics of people with MSA are comparable to internationally accepted norms, as MSA is seen as a disease predominantly presenting in people in their 40s–60s.^2^

The delay in diagnosis of MSA is similar to that experienced by people with other related conditions, where, due to the similarity in presentation, the condition is often misdiagnosed initially as Parkinson’s disease.^1 2^ Moreover, people with MSA may present in a context where neurological disease may not be considered, such as with sleep or urogenital or autonomic symptoms, and there is a need to ensure that all specialities are more aware of the possible diagnosis.^1^ Since 2022, the Movement Disorder Society has revised its diagnostic criteria and defined two levels of clinical diagnosis: clinically established and clinically probable MSA, as well as a proposed category of possible prodromal MSA.^11^ This may encourage neurologists to consider the diagnosis earlier, but it may also lead to issues as to how best to discuss a possible diagnosis with patients and their families.^6^

Even when diagnosed, there seems to be a reluctance to provide information about the disease and 41% have been given no information. This is often seen in other neurological diseases and the National Institute of Health and Care Excellence guidance on MND recommends discussion of the disease, its progression, the symptoms that may occur and the start of discussion for later care, including at the end of life.^12^ A similar approach, with time for discussion with a multidisciplinary team who are aware and knowledgeable about MSA, would be very helpful, allowing this discussion early after diagnosis and continuing throughout the disease progression.^1^ However, such a model does not currently routinely exist in the UK.

End-of-life care had been discussed by many of the respondents, and this had been found to be helpful by the majority. Only a minority of people with MSA (38%) had been able to discuss end-of-life care with an HCP and this may reflect the reluctance of professionals to undertake these discussions. A survey of movement disorders clinicians showed that although many clinicians wished to involve palliative care, there were concerns for a minority that discussion was uncomfortable and less necessary as neurological illness does not increase mortality or could lead to ‘loss of hope’.^13^

Specialist palliative care was provided to only a minority of respondents, although many had not been referred, and 41% of people with MSA felt that it was not required. There appeared to be barriers to the provision of specialist palliative care, including restricted periods of involvement and discharge from palliative care services if there were no active issues or issues accessing services. This has been seen elsewhere and a survey of European neurologists and palliative care specialists showed that less than 33% were seeing patients with MSA on a regular basis, compared with over 65% being involved in the care of MND.^7^ Movement disorder clinicians also reported that 27% of the palliative care services would not accept patients with MSA, although they did want to provide this care, either themselves or in collaboration with specialist palliative care services.^13^ There are ongoing debates about the availability and accessibility of people with neurological disease to specialist palliative care, with this group being disadvantaged compared with patients with cancer.^5 14^

The care received at the end of life was described by 65% of surviving carers as satisfactory, but 15% were unhappy with the care at the end of life. 21% did not feel that end-of-life care had been required, but this may reflect the number of carers who were surprised at the death. Death was often unexpected or was considered possible but not at the time of death. There are some who die a ‘sudden’ death,^15 16^ but many would have been expected and carers and families may not have been able to discuss the possible disease progression and the possibility of sudden death with professionals during the disease progression. Other studies have shown that patients may feel that they are not sick enough to discuss the issues or would prefer to concentrate on staying alive, although the majority (53%) did wish to discuss the end of life.^8^ This emphasises the need for wider multidisciplinary team involvement and discussion throughout the disease progression.^4^

ACP was undertaken within this group, but this was mainly in consideration of financial and care needs rather than specific plans for care and end-of-life care. This is similar to other studies where the discussion may occur, but advance directives are seldom prepared.^8^ Involvement of specialist palliative care may be helpful in encouraging and facilitating ACP, and potential triggers have been suggested for palliative care intervention and the ongoing discussion of goals of care—early in the disease trajectory when there is autonomic dysfunction and falls, mid-trajectory when speech disturbance is noticed and advanced stages.^17^ However, there has been increasing discussion of helping people with progressive disease plan ahead, rather than looking only at end-of-life care, looking ahead at the next stages in progression, the overall goals of care and the views of the person on care options.^18^,^20^ However, this may be more difficult with MSA, as communication may make later decision-making more difficult and earlier ACP may be necessary before the person feels prepared for these discussions.^19^ This again emphasises the need for ongoing care throughout the disease trajectory so that the person with MSA, and their family, can be fully involved in the setting of goals of care and the possible decisions that may need to be taken.

## Conclusions

People with MSA and their carers face many issues—physical, psychological and spiritual—and they should be able to discuss their concerns throughout the disease progression. More awareness of MSA and the issues that come with this diagnosis is needed, including increased collaboration with professionals, to enable earlier diagnosis and improved support throughout the disease trajectory.

## Data Availability

Data are avaialble on request.
